# Dietary supplement use among dietetics students at the University of KwaZulu-Natal

**DOI:** 10.4102/hsag.v24i0.1298

**Published:** 2019-09-26

**Authors:** Lynelda Pillay, Kirthee Pillay

**Affiliations:** 1 Department of Dietetics and Human Nutrition, College of Agriculture, Engineering and Science, University of KwaZulu-Natal, Pietermaritzburg, South Africa

**Keywords:** Dietary Supplements, Dietetics Students, UKZN, University Students

## Abstract

**Background:**

A dietary supplement is a product that aims to add nutritional value to the diet. University students are known to make use of dietary supplements to improve their academic performance, increase energy levels and promote overall general health. Based on assumption, students studying towards a nutrition-related degree may eat healthily and choose not to use dietary supplements. Alternatively, because of their interest in and exposure to nutrition, they may decide to use dietary supplements. However, there is a lack of published studies investigating the prevalence of dietary supplement use and reasons for use among South African university students studying towards a nutrition-related degree.

**Aim:**

The aim of this study was to assess the use of dietary supplements by dietetics students.

**Setting:**

University of KwaZulu-Natal (UKZN).

**Methods:**

A cross-sectional, descriptive study was conducted using a self-administered questionnaire.

**Results:**

Of the 139 participants, 23% (*n* = 32) used dietary supplements. There was a greater use by female students, those who lived at home and those registered for the Postgraduate Diploma in Dietetics. Reasons for using dietary supplements included the following: to strengthen the immune system (62.5%), to improve energy levels (56.3%) and to enhance physical health (50%). Cost (32.7%; *n* = 35), an adequate diet (22.4%; *n* = 24) and not necessary or waste of money (15%; *n* = 16) were reasons for not using dietary supplements. Most students (84.4%) made use of a multivitamin and mineral supplement.

**Conclusion:**

There was a low prevalence of dietary supplement use by UKZN dietetics students, with the high cost of supplements given as the main reason for non-use.

## Introduction

A dietary supplement is a product that intends to add nutritional value to the diet (Food and Drug Administration 2015). The use of dietary supplements is favoured among many countries, with a steady increase in use (Lieberman et al. 2015). The different supplements used include multivitamins, multiminerals, amino acids and individual vitamin and mineral supplements such as vitamin A, B-complex, C, magnesium and zinc. Dietary supplementation is also considered an important strategy in the treatment and prevention of chronic diseases such as cancer and coronary heart disease. Given the harmful effects of micronutrient deficiencies, dietary supplementation has been shown to improve quality of life (Suleiman et al. 2008); however, this is debatable.

Many groups, including female adults, the elderly, health professionals, gym goers, pregnant women, children under the age of 18 years and university students, particularly health science students, are known to make use of dietary supplements. Students are a group known to make use of dietary supplements to aid their academic performance (Aina & Ojedokun 2014; Steele & Senekal 2005). Specifically, students studying medical- and health-related degrees have been shown to have a higher prevalence of dietary supplement use (Aina & Ojedokun 2014). Some of the reasons cited for use of dietary supplementation by university students include recommendation from a doctor to improve health and immune status, supplement the current diet, improve energy levels, aid weight loss, increase muscle strength and promote overall general health (Aina & Ojedokun 2014; Lieberman et al. 2015).

A South African study found that students used supplements for ‘physical health’, ‘body conditioning’ and ‘dietary reasons’, ‘perceived dietary inadequacy’, ‘convenience’ and the belief that food contains an inadequate amount of nutrients. However, this study did not investigate dietary adequacy and intake (Steele & Senekal 2005). Some of the reasons cited by students for not using supplements included ‘cost’, ‘not important’ and ‘adequate diet’ (Driskell 1999). Although students may share a variety of interests and have similar lifestyles, their use of dietary supplements may differ from the general population (Lieberman et al. 2015).

Knowledge on nutrition was found to improve with education among students studying nutrition-related courses (Van der Kruk et al. 2013). However, it is not known if this exposure and knowledge on nutrition influenced them to make use of dietary supplements (Van der Kruk et al. 2013). There was an increased prevalence of supplement use by students studying nutrition or dietetics as they progressed with their studies (Van der Kruk et al. 2013). It is assumed that students studying a nutrition-related degree such as dietetics would be more likely to use dietary supplements to improve performance, nutritional status and overall health. However, there is a lack of published South African studies on this topic. Therefore, this study aimed to assess the use of dietary supplements by dietetics students at the University of KwaZulu-Natal (UKZN). The specific objectives were to determine the prevalence and reasons for use.

## Methods

### Study design

A cross-sectional study design was used to assess the use of dietary supplements by dietetics students at UKZN.

### Study population

The study population consisted of all students registered for either the Bachelor of Science in Dietetics degree or the Postgraduate Diploma in Dietetics at UKZN in semester 2 of 2017. At UKZN, students first complete a bachelor’s degree over 3 years, followed by the diploma which is also regarded as the fourth year of study. At the time of the study there were 156 dietetics students registered at UKZN, and all were invited to participate in the study. Students registered for Postgraduate degrees in Dietetics were not included in the study.

### Questionnaire

Data were collected using a self-administered questionnaire. The questionnaire was developed in English as this is the medium of instruction at UKZN and it was assumed that all students understood the questionnaire. The questionnaire was made up of both open and closed-ended questions and consisted of four sections. Section A obtained the socio-demographic data; section B covered lifestyle factors; section C obtained dietary data and section D obtained information on dietary supplementation. A pilot study was conducted prior to the main study, to validate the questionnaire. Ten students registered for qualifications other than dietetics on the Pietermaritzburg campus of UKZN were randomly approached and invited to participate in the pilot study. Pilot study participants read the information sheet and signed a consent form prior to participating. The pilot study determined that it took between 10 and 15 min to complete the questionnaire. However, no changes were made to the wording of the questions in the questionnaire. The questionnaire was also validated by the study supervisor and a statistician, who checked that the questions answered the objectives of the study and that they were presented in a logical manner, without any leading, ambiguous or confusing questions.

### Data collection

With permission from the lecturers in the Department of Dietetics and Human Nutrition, the researcher was allowed to address the dietetics students at the start of their lecture periods. The researcher explained the aims and objectives of the study and invited students to participate. Those who were interested in participating were given an information sheet and consent form. After reading the information sheet and signing the consent form, the questionnaire was issued to the student. The researcher was present during the entire time the questionnaire was being answered, to respond to questions or queries from the students. The students were not forced to participate in this study and were free to withdraw at any time. Data collection was conducted at UKZN, Pietermaritzburg campus and the Innovation Centre, UKZN, Durban for the Durban-based students registered for the Postgraduate Diploma in Dietetics. Out of the 156 registered dietetics students, 139 completed valid questionnaires were obtained, resulting in a response rate of 89%.

### Data analysis

All data entry was cross-checked for inconsistencies by the researcher. The Statistical Package for Social Sciences (SPSS) version 22 was used to analyse data. Data were summarised using frequencies and percentages. For comparison between categories, the Pearson chi-square test was used. A *p*-value of < 0.05 was considered significant.

### Sample characteristics

Table 1 shows the sample characteristics.

**TABLE 1:** Sample characteristics (*n* = 139).

Variables	*n*	%	Use of dietary supplement	
			Yes	No
**Age**				
18–19 years	35	25.2	4	31
20–22 years	60	43.2	13	47
23–25 years	30	21.6	11	19
> 25 years	14	10.1	4	10
**Gender**				
Male	36	25.9	3	33
Female	103	74.1	29	74
**Qualification**				
BSc in Dietetics	118	84.9	21	97
Postgraduate Diploma in Dietetics	21	15.1	11	10
**Year of study**				
First	33	23.7	6	27
Second	46	33.1	7	39
Third	39	28.1	8	31
Fourth	21	15.1	11	10
**Where students lived during the term**				
Home	36	25.9	14	22
University residence	48	34.5	6	42
Commune	32	23.0	4	28
Flat or apartment	21	15.1	7	14
Other	2	1.4	0	2
**Source of funding**				
Parents or guardian	49	35.3	15	34
Bursary or scholarship	22	15.8	10	12
Student loan	4	2.9	1	3
Financial aid	59	42.4	5	54
Other	5	3.6	1	4

Of the 139 participants, 74.1% (*n* = 103) were female students and 25.9% (*n* = 36) were male students. Most (43.2%; *n* = 60) were between 20 and 22 years old, followed by 25.2% (*n* = 35) who were between 18 and 19 years old. A total of 118 (*n* = 84.9%) were registered for the BSc Dietetics degree, while 15.1% (*n* = 21) were registered for the Postgraduate Diploma in Dietetics. Thirty-three (23.7%) were in first year, followed by 33.1% (*n* = 46) in second year and 28.1% (*n* = 39) in third year. About 35% (*n* = 48) lived at the university residence, followed by home (25.9%; *n* = 36) and a commune (23.0%; *n* = 23). The main source of funding was financial aid (42.4%; *n* = 59), followed by parent or guardian (35.3%; *n* = 49) (Table 1).

### Dietary supplement use

Table 2 shows that 23% (*n* = 32) of the participants used dietary supplements, while 77% (*n* = 107) did not. Significantly more women (20.9%; *n* = 29) used dietary supplements compared with men (2.2%; *n* = 3) (*p* = 0.018) (Table 1). Students who lived at home were more likely to use dietary supplements (46.9%; *n* = 15) (*p* = 0.008), and the highest use of dietary supplements was observed among the students registered for the Postgraduate Diploma in Dietetics, equivalent to a fourth year of study (*p* = 0.006). More than half of the participants (62.5%; *n* = 20) indicated that they used dietary supplements to strengthen their immune system, followed by improving energy levels (56.3%; *n* = 18) and to enhance physical health (50.0%; *n* = 16). Cost (32.7%; *n* = 35), an adequate diet (22.4%; *n* = 24) and not necessary or waste of money were statistically significant reasons for not using dietary supplements (*p* < 0.05) (Table 2).

**TABLE 2:** Dietary supplement use.

Variable	*n*	%
**Use of dietary supplements (*n* = 139)**		
Yes	32	23.0
No	107	77.0
**Reasons for using a dietary supplement (*n* = 32)**		
Inadequate dietary intake	10	31.3
Enhance sports performance	2	6.3
Enhance memory and concentration	14	43.8
Strengthen the immune system	20	62.5
Improve energy levels	18	56.3
Enhance physical health	16	50.0
Medical condition requires supplements	8	25.0
Based on nutrition knowledge or background	12	37.5
Other	1	3.1
**Reasons for not using a dietary supplement (*n* = 107)**		
Supplements are expensive	35	32.7
Diet is adequate	24	22.4
Not necessary or waste of money	16	15.0
Undesirable side effects	8	7.5
Unsure about supplements	15	14.0
Supplements are not effective	1	0.9
Unhealthy to use a supplement	1	0.9
Other	7	6.5

According to Table 3, a multivitamin and mineral supplement was the most common type of supplement used (84.4%; *n* = 27), followed by minerals only (18.8%; *n* = 6), vitamins only (15.6%; *n* = 5) and herbal supplements (15.6%; *n* = 5). Supplement users mostly obtained their supplements from a pharmacy (62.5%; *n* = 20), followed by a health store (46.9%; *n* = 15). Only 12.5% (*n* = 4) of supplement users obtained their supplements from a hospital or clinic. Of those who used supplements, 65.6% (*n* = 21) used them every day, while 25% (*n* = 8) used them 2–6 times a week. R151–R500 was the most common amount spent on supplements monthly, followed by less than R150 (28.1%; *n* = 9). Approximately 19% (*n* = 6) of supplement users obtained their supplements for free (Table 3).

**TABLE 3:** Types of supplements, source and frequency of use (*n* = 32).

Variable	*n*	%
**Types of supplements used**		
Multivitamin and mineral	27	84.4
Minerals only	6	18.8
Vitamins only	5	15.6
Amino acids or protein	3	9.4
Essential fatty acids	3	9.4
Herbal supplements	5	15.6
Other	1	3.1
**Source of supplements**		
Doctor	4	12.5
Hospital or clinic	4	12.5
Health store	15	46.9
Pharmacy	20	62.5
**Frequency of use**		
Sporadically (every 6 months)	3	9.4
Once a week	0	0
Two to 6 times a week	8	25.0
Every day	21	65.6
**Money spent on supplements (monthly)**		
Free	6	18.8
< R150	9	28.1
R151–R500	15	46.9
> R500	2	6.3

**FIGURE 1:**
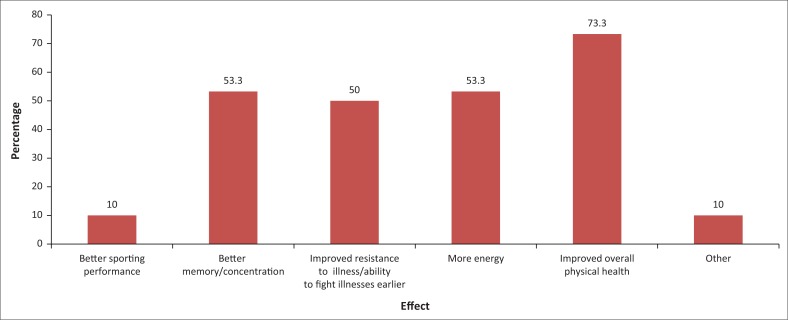
Effect of dietary supplement use.

Figure 1 indicates that 73.3% of supplements users indicated that they had experienced an overall improvement in physical health after using dietary supplements, followed by better memory or concentration (53.3%) and more energy (53.3%). Furthermore, half of the users reported an improved resistance to illness or ability to fight illnesses earlier (Figure 1).

### Ethical consideration

Ethical approval was obtained from the University of KwaZulu-Natal, Humanities and Social Sciences Research Ethics Committee (reference number: HSS/00358/017M). The Registrar of the University of KwaZulu-Natal also approved the study. Participants gave written consent prior to participating and confidentiality was maintained at all times. They were also informed that their participation was voluntary and that they were free to withdraw from the study at any time, without any negative consequences.

## Discussion

This study aimed to determine the prevalence of dietary supplement use among UKZN dietetics students and the reasons for use. Only 23% of the sample used dietary supplements, and of these 65.6% used them daily. Dietary supplements were mainly used for strengthening the immune system and improving energy levels. Multivitamins (vitamins and minerals) were the most commonly used supplement. Most users obtained their supplements from a pharmacy and spent between R151 and R500 monthly. Improved overall physical health, better memory and concentration and improved energy levels were some of the effects reported from the use of dietary supplements.

In the current study, only 32 students (23%) reported use of dietary supplements, while 76.9% (*n* = 107) indicated that they were not using dietary supplements at the time of the study. This indicated that there was a low prevalence of use of dietary supplements in this sample. This is in contrast to a study by Van der Kruk et al. (2013) on 568 Dutch nutrition and dietetics students, which found an increasing prevalence of supplement use by students studying nutrition or dietetics (Van der Kruk et al. 2013). This could possibly be because of the sample size of the current study being smaller, with only 139 students. Suleiman et al. (2008) also reported a low prevalence of dietary supplement use among health science and non-health science students in Jordan.

The main reasons cited for using supplements included strengthening the immune system, improving energy levels, enhancing physical health and enhancing memory and concentration. This indicates that students were concerned about their health and wanted to achieve optimal health, to aid their academic performance. This was supported by other studies, which also cited similar reasons (Aina & Ojedokun 2014; Lieberman et al. 2015; Sharma, Adiga & Ashok 2014; Suleiman et al. 2008). The students, in this study, cited expense, having an adequate diet, ‘deem it unnecessary/waste of money’, undesirable side effects or consequences and unsure about dietary supplements, as their reasons for not using a dietary supplements. These reasons are similar to those cited by students from previous studies (Kostka-Rokosz et al. 2015; Steel & Senekal 2005; Tian, Ong & Tan 2009). The fact that 32.7% cited expense as a reason for not using supplements is in keeping with the fact that 42.4% were on financial aid as a source of funding. Given the financial limitations that these students have, dietary supplements may be viewed as a luxury that they cannot afford.

There was a significant relationship between gender and the use of dietary supplements, with more women using dietary supplements compared with men. This was consistent with other studies that reported a higher use of dietary supplements among female participants (Bailey et al. 2010; Kim et al. 2014; Kishiyama et al. 2005; Spencer, Bendich & Frank 2006; Steele & Senekal 2005; Van der Horst & Siegrist 2011). The higher dietary supplement use among women could be because women are generally more concerned about their overall health and well-being than men. In this study, students who lived at home used dietary supplements more than those who lived elsewhere. This could be because the dietary supplements were likely purchased by parents at home and were more readily available. This study also found that the highest use of dietary supplements was observed among the fourth-year students. This is consistent with findings from Van der Kruk et al. (2013), who also reported that more fourth-year dietetics students consumed more dietary supplements compared with first-year students. This could be because of the fact that fourth-year students have more nutrition knowledge on the benefits of dietary supplements compared with first-year students. Thus, they were able to make a more informed decision regarding supplementation. It could also be that with experience, senior students have come to realise the role of dietary supplements in enhancing academic performance and optimising overall nutritional status and health.

The majority of the supplement users in the current study (84.4%) used a multivitamin and mineral supplement, followed by minerals only (18.8%) and vitamins only (15.6%), which is in line with previous studies (Aina & Ojedokun 2014; Kostka-Rokosz et al. 2015; Lieng et al. 2008; Suleiman et al. 2008). A multivitamin and mineral supplement may be a popular choice of supplement among students as they provide a variety of vitamins and minerals in one tablet, which is also convenient. More than half of the supplement users obtained their dietary supplements from the pharmacy, while 43.8% obtained their dietary supplement from a health store. Tian et al. (2009) reported similar results with the health food store (50.8%; *n* = 32) and the pharmacy (20.6%; *n* = 13), being the most common places from where dietary supplements were purchased. In this study, 65.6% of the supplement users reported taking their dietary supplement daily. This was more frequent compared to a study by Sharma et al. (2014), where 58.9% of participants consumed dietary supplements daily. In this study, participants who used dietary supplements sporadically did so because of forgetfulness and because they were only used when stressed. This is supported by Steele and Senekal (2005), who cited ‘when stressed’ (30.8%), ‘when tired’ (23.1%) and forgetting (16.7%) as the main reasons for the sporadic use of dietary supplements. In the current study, approximately 47% of supplement users spent between R151 and R500 per month on supplements, which may be a significant amount of money for a student to spend. This indicates that these users valued their health and may have prioritised spending on dietary supplements, over other items. A small number of users (18.8%) obtained their dietary supplements for free. These were likely to have been obtained from the university campus health clinic or government hospital or clinic, as they are issued for free. It may be worth investigating if students would be more likely to use dietary supplements if they were cheaper or free, as cost was a main reason for non-use.

In the current study, approximately 73.3% of supplement users indicated that they had experienced an overall improvement in physical health after using dietary supplements. Other results achieved included improved energy levels and better memory or concentration. Furthermore, half of the supplement users reported an improved resistance to illness or ability to fight illnesses earlier. Kostka-Rokosz et al. (2015) also reported that 49% of nursing students and 21% of pharmacy students were satisfied with the efficacy of their dietary supplement. The positive health outcomes associated with the use of dietary supplements among students may encourage long-term use of the supplements.

## Study limitations and recommendations

This study was only conducted on dietetics students registered at UKZN, which does not allow for generalised conclusions on dietetics students in general. Future studies should be conducted on dietetics students across other South African universities where the programme is offered, to determine whether students studying towards a nutrition-related qualification are more likely to use dietary supplements.

## Conclusion

Contrary to the assumption that students studying towards a nutrition-related degree would be more likely to use dietary supplements because of their interest in and exposure to nutrition, there was a low prevalence of dietary supplement use among dietetics students at UKZN. The most commonly used dietary supplement was a multivitamin and mineral supplement. Expense was one of the main reasons cited for not using dietary supplements, while those who used supplements did so for strengthening the immune system, improving energy levels and enhancing physical health. Dietary supplement users reported that they experienced improved physical benefits, including better memory and concentration and more energy. Given the poor dietary habits of students and limited finances to purchase a variety of foods, dietary supplements may have a role to play in improving nutritional status. Students should also have access to reliable and accurate information on the need and use of dietary supplements, to prevent unnecessary and unsafe supplementation.
